# Traditional Chinese Medicine Pulse Diagnosis on a Smartphone Using Skin Impedance at Acupoints: A Feasibility Study

**DOI:** 10.3390/s20164618

**Published:** 2020-08-17

**Authors:** Kun-Chan Lan, Gerhard Litscher, Te-Hsuan Hung

**Affiliations:** 1 Department of CSIE, National Cheng Kung University, Tainan 701, Taiwan; crab@isu.edu.tw; 2 School of Chinese Medicine, China Medical University, Taichung 404, Taiwan; 3 Research Unit of Biomedical Engineering in Anesthesia and Intensive Care Medicine, Research Unit for Complementary and Integrative Laser Medicine, and Traditional Chinese Medicine (TCM) Research Center Graz, Medical University of Graz, 8036 Graz, Austria

**Keywords:** pulse diagnosis instrument, photoplethysmography, galvanic skin response, resonance theory, smartphone

## Abstract

In traditional Chinese medicine (TCM), pulse diagnosis is one of the most important methods for diagnosis. A pulse can be felt by applying firm fingertip pressure to the skin where the arteries travel. The pulse diagnosis has become an important tool not only for TCM practitioners but also for several areas of Western medicine. Many pulse measuring devices have been proposed to obtain objective pulse conditions. In the past, pulse diagnosis instruments were single-point sensing methods, which missed a lot of information. Later, multi-point sensing instruments were developed that resolved this issue but were much higher in cost and lacked mobility. In this article, based on the concept of sensor fusion, we describe a portable low-cost system for TCM pulse-type estimation using a smartphone connected to two sensors, including one photoplethysmography (PPG) sensor and one galvanic skin response (GSR) sensor. As a proof of concept, we collected five-minute PPG pulse information and skin impedance on 24 acupoints from 80 subjects. Based on these collected data, we implemented a fully connected neural network (FCN), which was able to provide high prediction accuracy (>90%) for patients with a TCM wiry pulse.

## 1. Introduction

For several thousands of years, the pulse diagnosis has been one of the important diagnostic methods used in traditional Chinese medicine (TCM). A pulse can be felt by applying firm fingertip pressure to the skin at sites where the arteries travel near the surface of the skin. The pulse diagnosis has become a necessary procedure in clinical diagnosis not only for Chinese medicine practitioners but also in several areas of Western medicine. In the past, Chinese medicine practitioners relied on their accumulated experience and intuition, where the sensitivity of different physicians varied. It was difficult to transfer personal experiences to other practitioners. Currently, through a combination of medical expertise and digital signal processing technologies, TCM pulse-measuring instruments not only greatly improve reproducibility, but also instantly visualize and analyze the measured pulse profile. There are about 29 pulse types in a TCM pulse diagnosis, including the floating pulse, scattered pulse, hollow pulse, deep pulse, hidden pulse, firm pulse, slow pulse, moderate pulse, swift pulse, surging pulse, thready pulse, long pulse, short pulse, feeble pulse, weak pulse, faint pulse, replete pulse, slippery pulse, stirred pulse, unsmooth pulse, wiry pulse, tight pulse, tympanic pulse, soggy pulse, irregularly intermittent pulse, irregular pulse, irregular-rapid pulse, and intermittent pulse [1,2]. The key point of pulse-measuring instruments is to obtain important information from the pulse in order to distinguish between different pulse types. A comprehensive analysis of the pulse types can lead to an understanding of a disease and provide a basis for the dispensing of prescriptions [3].

While many pulse-measuring devices have been developed based on different sensing techniques, such as piezoresistive, photoelectric, and piezoelectric methods [4,5,6,7], they are mostly single-probe sensors that convert the spatial variations of pulses into a simple output of electrical signals. These single-point sensing devices can only obtain limited information because they cannot reflect the detailed changes in the pulse. Physiologically speaking, the TCM pulse type is “felt” by many different receptors on the fingertip of a TCM practitioner. Each of these receptors can be considered to be a single sensor. Intuitively, data obtained from only a single pulse sensor will not have enough information to correctly model the “pulse feeling” based on data from multiple neural receptors on the fingertip.

Chung et al. [8] recently developed a multi-point sensing instrument based on a 3 × 4 sensor array. While their device is able to identify the important characteristics of the wrist radial artery, including strength, rate, length, width, and trends in pulse conditions, using three-dimensional pulse mapping (3DPM) [8] of the pulse and simulating the sensation of actual touch, it is too costly and requires running its sophisticated signal processing algorithms on a PC.

To address the shortcomings of these prior studies [5,6,7,8], in this work, we propose a smartphone-based sensor system for TCM pulse diagnosis built on the concept of “sensor fusion.” The idea of sensor fusion is to combine sensory data from multiple sources such that the resulting information is more complete than would be possible using these sources individually. To be more specific, we consider additional information from recording the conductivity of acupuncture points to complement the use of a single-probe pulse sensor when inferring the TCM pulse type.

Acupuncture points have been proven to have distinct electrical properties. These properties include increased conductance, reduced impedance and resistance, increased capacitance, and elevated electrical potential compared to adjacent non-acupuncture points [9]. Based on these properties, skin impedance at acupuncture points (APs) has been used as a diagnostic aid for various diseases for more than 50 years [9]. In this work, three reasons motivated the use of information about AP skin impedance to assist the inference of TCM pulse type. First of all, both TCM pulse and AP impedance are basically biological features that reflect the conditions of the body. While these two biomarkers are different, hypothetically, their data distributions could be correlated when they are measured at the same time for a given physical state. Theoretically, if two data distributions, say *p* and *A*, are correlated, augmented data from *A* (e.g., acupoint conductance) may be helpful as a way to implicitly learn about *p* (e.g., pulse characteristics) [10]. Second, skin impedance information related to APs can be non-intrusively and easily collected through a low-cost galvanic skin response (GSR) sensor. Finally, from the perspective of sources of sensing, there are many acupoints on the body, which in aggregation can provide rich information about the condition of the human body (considering the amount of data collection time, in this work, we used only 24 acupoints).

Our contribution in this work is threefold. First, we designed and implemented a low-cost smartphone-based sensor system consisting of one photoplethysmography (PPG) sensor and one galvanic skin response (GSR) sensor to infer TCM pulse type based on the concept of sensor fusion. Second, as a proof of concept, we collected single-point PPG pulse information and GSR data for 24 acupoints from 80 subjects with a “wiry pulse,” a commonly seen TCM pulse type usually found in patients with liver diseases. Eighteen features were computed from PPG signals and twenty-four features were extracted from GSR data, respectively. We collected five-minute PPG measurements from each subject. A sliding-window (with a window size of 10 s) approach was implemented to segment PPG data for computing the eighteen features. The GSR data for each acupoint were sampled for five seconds and the median of the sampled GSR was recorded. These data were used to verify our hypothesis suggesting a correlation between TCM pulse and acupoint impedance. Finally, based on these collected data, we implemented a binary classifier based on a fully connected neural network (FCN), which was able to accurately predict the wiry pulse. To the best of our knowledge, this is the first low-cost (without considering the smartphone, the cost of our system is under $200) portable sensor system that can correctly infer the TCM pulse type. As compared to prior work by Chung et al. [6], our system is more affordable for use by TCM practitioners.

The remainder of this paper is organized as follows: We present our methods in Section 2 and evaluate the proposed system in Section 3. Finally, we conclude this paper and provide suggestions for future work in Section 4.

## 2. Method

**Figure 1:**
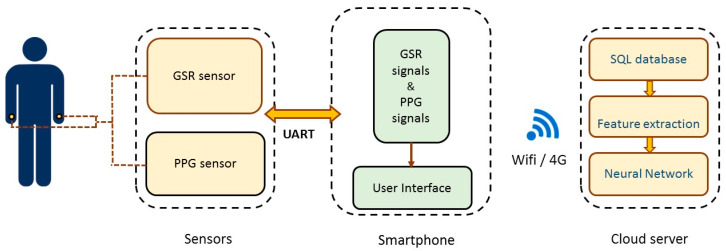
System architecture.

The hardware architecture of the proposed system is shown in Figure 1. It is divided into three parts, including one PPG sensor and one GSR sensor, a smartphone, and a cloud server. PPG is a signal that is recorded by using a skin area to absorb light energy according to the light sensing element. Since the hemoglobin in the blood absorbs light, the amplitude of the PPG signal changes proportionally with the blood flow in and out of tissue. In this study, we used the GSR sensor to record the skin impedance of acupoints. In TCM, it is generally believed that acupoints are the reaction points that reflect health conditions and pathological changes in the human body. Yoshio Nakatani [9] found that patients with various visceral diseases had specific skin resistance response patterns at some particular reaction points called Ryodoraku points. Incidentally, these Ryodoraku points appear to be at the same location as 24 known TCM acupoints (as shown later in Table 1).

The sensor platform first collects GSR and PPG signals from the subject. These sensor data are sent through the OTG (On-The-Go) cable to the smartphone and then transmitted wirelessly to the cloud server to predict the TCM pulse type using an artificial neural network.

### 2.1. Sensors

**Figure 2:**
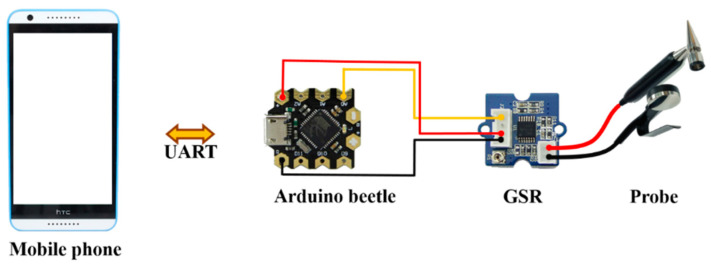
Wiring diagram of the GSR (galvanic skin response) sensor.

Figure 2 shows the wiring diagram of the GSR (galvanic skin response) sensing unit, which is comprised of an Arduino beetle board, a GSR module, and a probe. A mobile phone is used to transmit the GSR data to the cloud as well as to provide 5 V power to the Arduino beetle through a USB OTG. The GSR module (Seeed Studio, Shenzhen, China) consists of a bridge circuit and an amplifier. The bridge circuit is used to measure resistance. Its working principle is similar to that of a potentiometer. After the measured resistance is converted to a voltage through the bridge circuit, the amplifier amplifies the signals to the desired magnification. The amplified signals are read by the ADC (analogue digital converter) on the Arduino beetle and then relayed to the phone over UART (universal asynchronous receiver transmitter). Given that most phones usually do not have externally available UARTs, a USB-serial converter is employed for the communication between the phone and the Arduino beetle.

The wiring diagram of the PPG (photoplethysmography) sensor module is similar to the design of the GSR sensor. The PPG pulse sensor is connected to an Arduino beetle board, and we utilize the smartphone to read PPG data from the beetle board as well as to power the board. In this work, 5-minute PPG measurements were collected from the wrist of the user. The GSR data were collected from 24 acupoints based on the Ryodoraku points proposed by Dr. Yoshio Nakatani [9], and each acupoint was sampled for 5 s.

### 2.2. Smartphone

**Figure 3:**
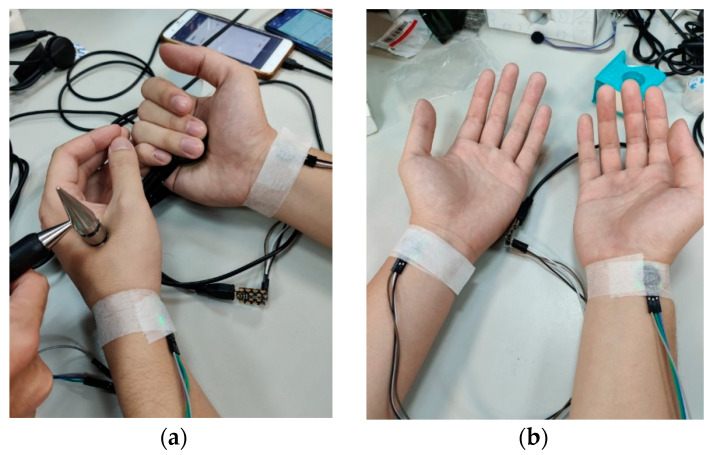
Placement of the (**a**) GSR and (**b**) PPG sensors.

The proposed system was implemented on the Android platform. Both the control packets and data packets were sent over UART between the phone and the PPG/GSR sensor. Note that, considering the resolution of the ADC on the Arduino beetle board is 10 bit while the packet size of UART is only one byte, we used a simple LZ-based method to compress the sensor data into one byte. During the measurements, as shown in Figure 3a, one end of the GSR probe was put on the palm while the other end was placed on the targeted acupuncture point. For the PPG measurements, the sensor was placed on the “Guan” position, which is adjacent to the styloid process of the radius bone (at the medial portion of the bulge of the prominent bone proximal to the palm) [11], following the practice used in traditional Chinese medicine. We used adhesive tape to wrap the PPG sensor around the wrist, as shown in Figure 3b, to reduce movement artifacts during the measurements. The sample rates for the PPG and GSR sensor were 25 Hz and 200 Hz, respectively. For the PPG, we collected all of the raw data. We only recorded the median of the GSR measurements for each acupuncture point.

A simple user interface was implemented on the phone that allowed the user to start/stop the sensor data collection and view the obtained signals in real time. The measured data were first stored in the phone and then transmitted to the cloud server at the end of each measurement process.

### 2.3. Cloud Server

**Figure 4:**
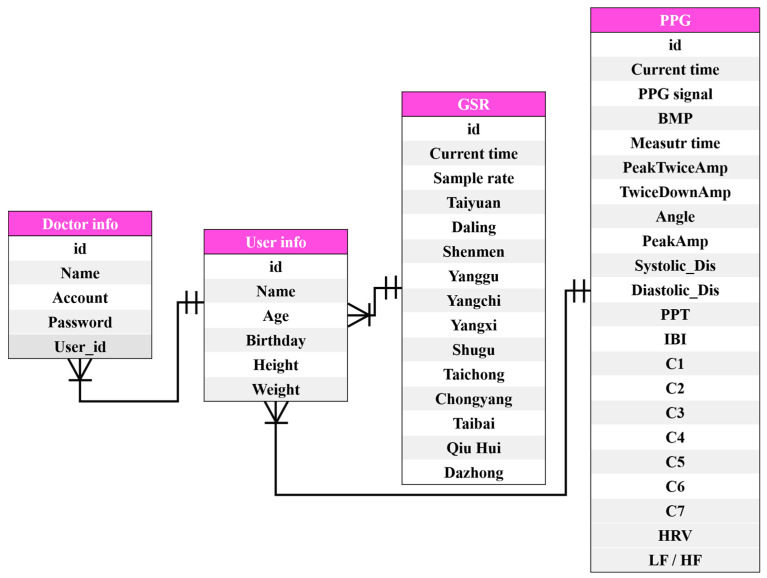
ER model of the SQL tables.

We used MySQL to store the GSR and PPG signals (including 18 features computed from the PPG signals (which will be discussed shortly). The ER (entity-relationship) model of the SQL tables is shown in Figure 4.

To predict the pulse type, we utilized forty-two features computed from PPG and GSR data (collected at various acupoints), as listed in Table 1.

**Figure 5:**
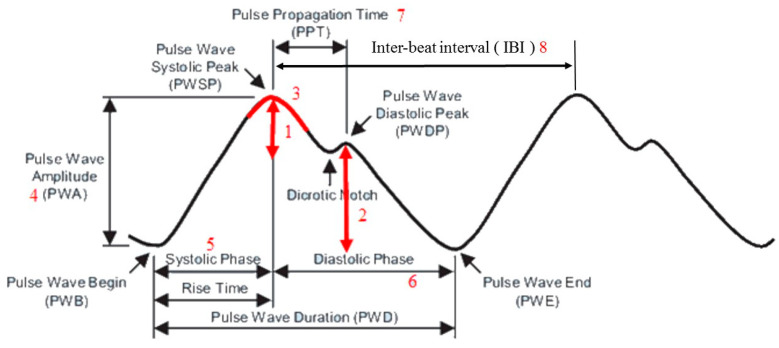
Time-domain features of the PPG signal.

**Figure 6:**
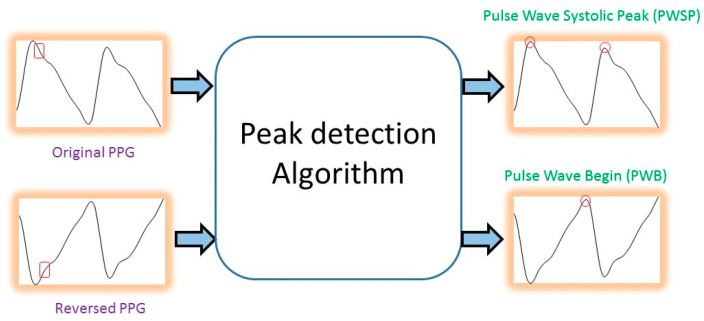
Detection of the pulse wave systolic peak (PWSP) and pulse wave begin (PWB).

Eighteen features were computed from PPG signals. These features consisted of 8 time-domain features and 10 frequency-domain features, as shown in Figure 5 and Figure 6, respectively. These features were selected based on an extensive survey of common features used in previous studies related to pulse diagnosis [4,5,6,7,8,9,12,13,14,15,16,17,18]. We implemented a simple PMAF [19] method to remove motion artifacts and noises in the PPG signals before computing the time- and frequency-domain features of the PPG data. The PMAF was based on the quasi-periodicity of the PPG signals, which first segments the PPG signal into periods and then resamples each period [20].

After removing the noise from the PPG waves, we first considered the three feature points for the time-domain features: the pulse wave systolic peak (PWSP), the pulse wave begin (PWB), and the pulse wave diastolic peak (PWDP), as shown in Figure 5. Once these feature points were located, the eight time-domain features shown in Figure 5 could be computed accordingly. To detect the pulse wave systolic peak (PWSP), we implemented an algorithm based on an event-related moving average [20]. This method consists of three stages: signal pre-processing (including bandpass filtering and squaring), generation of blocks of interest using two moving averages, and classification-based adaptive thresholding. The location of the pulse wave begin (PWB) can be detected using the same algorithm by changing the input to the reversed PPG signals (so that PWB becomes the maxima in the signals, as shown in Figure 6).

To detect diastolic peaks, we first computed the second-order derivates of the PPG waveforms, which were then smoothed using a moving average filter. Generally speaking, the largest minima within the second-order derivative waveform correspond to the systolic peaks, and the minima following these typically correspond to the diastolic peaks. Once the locations of the systolic peaks were identified, as above, we could perform peak detection on the inverted second-order derivative waveform to find the local maxima corresponding to the diastolic peak. The timing of the diastolic peak was then recorded as the timing of the minima following the systolic peak in each PPG wave.

Once the pulse wave systolic peak (PWSP), the pulse wave begin (PWB), and the pulse wave diastolic peak (PWDP) were identified, we could then compute the eight time-domain features, as shown from Equation (1) to Equation (8). Specifically, the location of the PWSP, PWB, and PWDP were given a 2D coordinate (x,y), where x is the amplitude of the feature point, and y is the timing in the signals. For example, PWSP(x,y) refers to the 2D coordinate of PWSP, and PWSP(x) indicates the x coordinate of PWSP. Feature 1, Feature 2, and Feature 4 could be computed using the basic Pythagorean theorem. The PWSP angle (Feature 3) was estimated by first connecting the PWSP with the sampled point before and the sampled point after into a triangle. The PWSP angle could then be computed using the inverse trigonometric function.

(1) $$Feature\;1=\sqrt{\left(\mathrm{PWSP}(x,y)-\mathrm{PWDP}(x,y){)}^{2}-(\mathrm{PWSP}(x)-\mathrm{PWDP}(x)\right)}$$

(2) $$Feature\;2=\;\sqrt{\left(\mathrm{PWDP}(x,y)-\;\mathrm{PWE}(x,y){)}^{2}-(\mathrm{PWDP}(x)-\mathrm{PWE}(x)\right)}$$

(3) $$Feature\;3=\;∠C=\;arccos\frac{a^{2}+b^{2}-c^{2}}{2ab}$$

(4) $$Feature\;4=\sqrt{\left(\mathrm{PWSP}(x,y)-\mathrm{PWB}(x,y){)}^{2}-(\mathrm{PWSP}(x)-\mathrm{PWB}(x)\right)}$$

(5) Feature 5= PWSP(x) − PWB(x)

(6) Feature 6= PWSP(x) − PWE(x)

(7) Feature 7= PWSP(x) − PWDP(x)

(8) Featreu 8= next PWSP(x)−PWSP(x)

The frequency-domain features were based on the resonance theory proposed by Wang [13]. He found that the intensity of the resonance waves of different frequencies and the physiological and pathological changes of different organs have a relationship [21]. Based on several animal and clinical observations, Wang claimed that C0 is the total load of the heart in a cardiac cycle. There are corresponding relationships between different harmonics and TCM meridians (as shown in Table 1). In this work, we collected 5 min PPG measurements from each subject. A sliding-window (with a window size of 10 s) approach was implemented to segment the PPG data for the purpose of computing the frequency-domain features. An FFT (fast Fourier transformation) was used to convert the PPG signals to frequency-domain information for each 10 s segment. Based on the subject’s heart rate calculated in each window, we computed the base frequency and found the harmonic of the base frequency from C1 to C10, as shown in Table 1.

**Figure 7:**
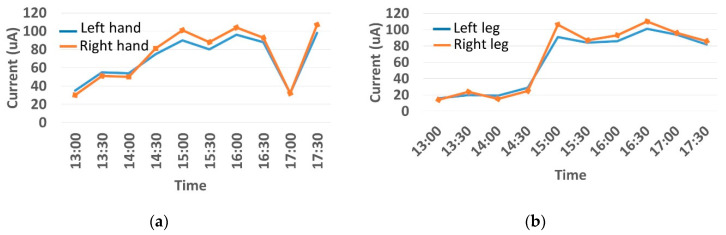
(**a**) The correlation between the Taiyuan acupoints (left hand vs. right hand). (**b**) The correlation between Taichong acupoints (left leg vs. right leg).

The features taken from the GSR data included skin impedance for 12 acupoints, including Taiyuan (LU9), Daling (PC7), Shenmen (HT7), Yanggu (SI5), Yangchi (TE4), Yangxi (LI5), Shugu (BL65), Taichong (LV3), Chongyang (ST42), Taibai (SP3), Chiu Hsi (GB40), and Dazhong (KD4), as shown in Table 1. The selection of these acupoints was based on the Ryodoraku points proposed by Dr. Yoshio Nakatani [9]. According to traditional Chinese medicine (TCM), these acupoints are present on both the left side and the right side of the body, and they are symmetrical. (Incidentally, as a side observation, based on measurements from 20 volunteers, we found the skin impedance taken from the acupoint on the left side of the body had a strong correlation with that from the same acupoint on the right side of the body, as in the examples shown in Figure 7.) Nevertheless, in this study, we used the 24 acupoints from both sides of the body in our experiments.

Finally, with the features extracted from the PPG and GSR data, we trained a simple neural network (NN) model based on a 5-fold validation scheme (i.e., 80% of the data were used for training, and 20% were used for testing) to predict the TCM pulse type. Specifically, we implemented a fully connected neural network (FCN), for which the inputs were the PPG and GSR data. The input layers had 42 neurons (18 of them were extracted PPG features and the other 24 were from the GSR data). The output layers had a single output that indicated the probability of predicting a specific pulse type. Three hidden layers with 30 neurons in each layer were employed in the experiments. In this study, we focused on the prediction of a “wiry pulse,” which is a commonly seen pulse type usually observed in people who are stressed or have liver-related diseases. To obtain the ground truth for the pulse type, three independent, experienced TCM doctors were responsible for the subject selection. A subject was only included into this study if he/she was diagnosed with the same pulse type by all three TCM doctors.

## 3. Results

Before we reported our results on TCM pulse type classification, we first validated the PPG and GSR sensor devices employed in this work against some commercial products. Next, as a proof of concept, we recruited 80 subjects (among them, 40 subjects had a “wiry pulse” based on agreement among three TCM doctors) with the goal of determining if the proposed sensor-fusion system could predict the same results agreed upon by the TCM doctors.

### 3.1. Validation of PPG/GSR Sensor Devices

**Figure 8:**
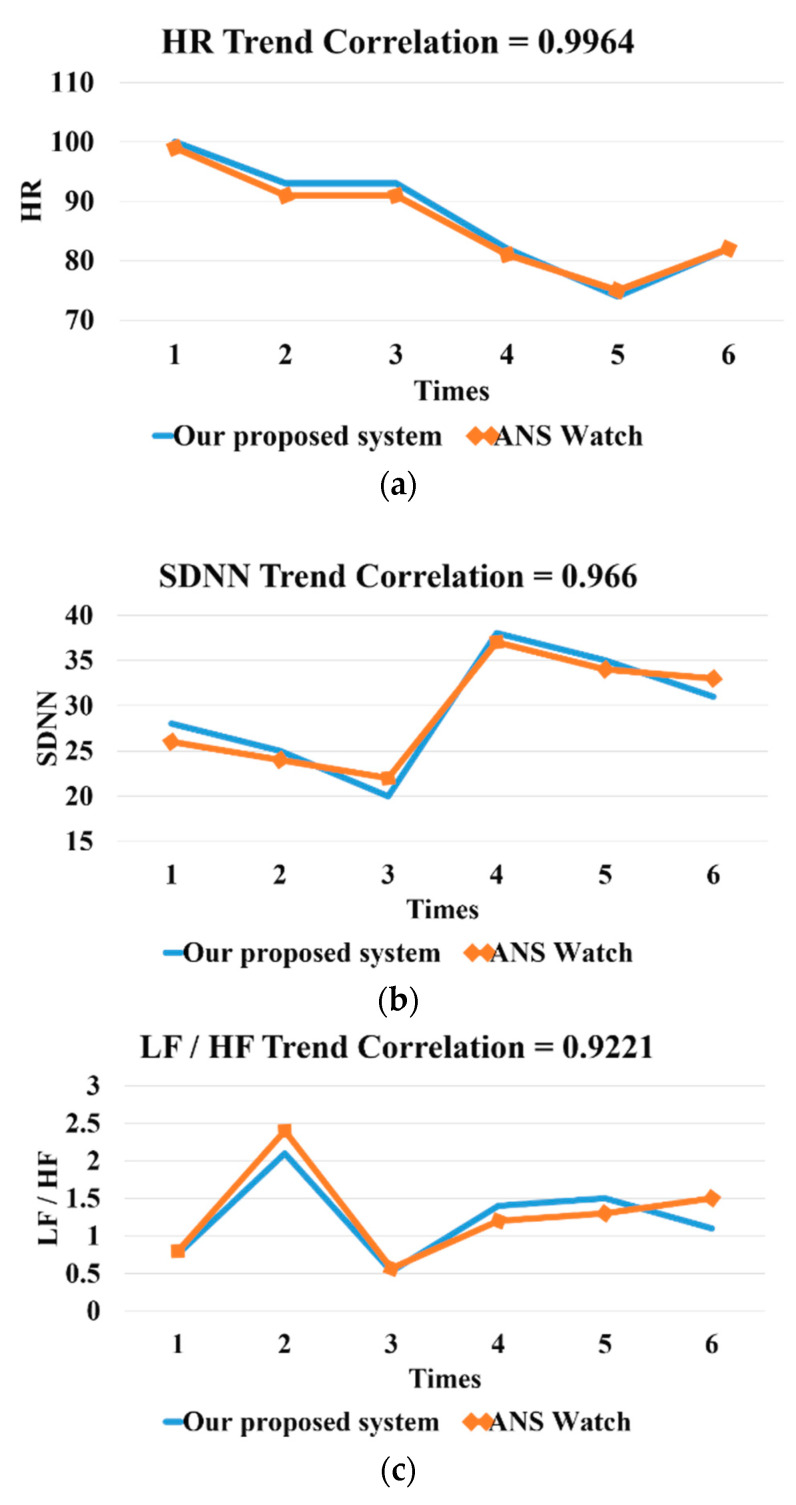
(**a**)The HR (heart rate) correlation. (**b**) The HRV (SDNN) correlation. (**c**) The LF/HF ratio correlation.

For the PPG comparison, we used a commercial product called ANSwatch (Model TS-0411) [22] as our benchmark. We compared three metrics, including heart rate, heart rate variability (SDNN), and LF/HF, obtained from ANSwatch and our PPG sensor device. We found a strong correlation (above 90%) between ANSwatch and our device, based on one of the subjects, as shown in Figure 8.

**Figure 9:**
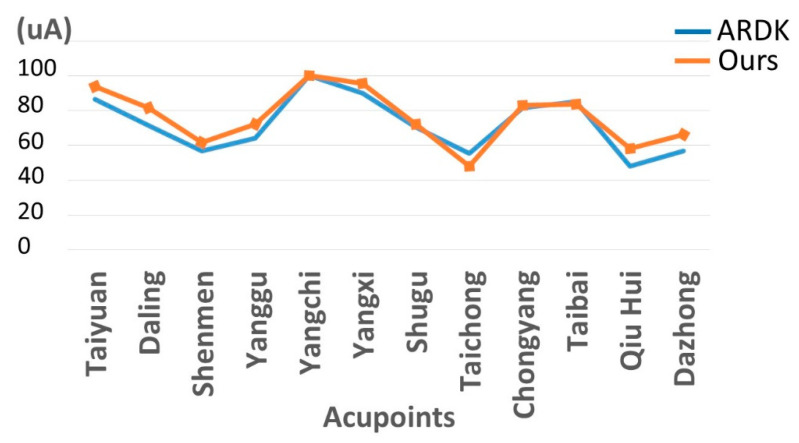
Comparison of the proposed GSR sensor with the Automatic Reflexodiagnostic Komplex (ARDK).

For validation of the proposed GSR sensor device, we used a commercial device called ARDK^®^ (Automatic Reflexodiagnostic Komplex; AleXorA BioMedical Technologies Inc. Quezon City, Philippine) [23] as our benchmark. In addition, we also validated the output of our GSR device with a multi-meter (CHY-36C). Four resisters were used (their resistances were 384 kΩ, 806 kΩ, 3.52 MΩ, 990 KΩ, and 3.81 MΩ, respectively), and our GSR device output the same readings as those from the multi-meter when measuring these resisters. When measuring the acupoints, the output of the proposed GSR device was consistent with the ARDK output (the correlation on average, based on 20 participants, was higher than 95%), as shown in the example in Figure 9.

### 3.2. Prediction of the Wiry Pulse

In this study, we recruited 80 subjects. Among them, 40 patients had a “wiry pulse,” and the others did not (among them, 38 had a “normal pulse” and 2 had a “slippery pulse”). The identification of pulse type for each subject was based on a joint agreement of three different TCM doctors. The sampling rate of the GSR sensor was 200 Hz, and that of the PPG sensor was 25 Hz. The duration of the PPG measurements was five minutes. A sliding-window (with a window size of 10 s) approach was implemented to segment the PPG data before feeding the data into the neural network. The total number of PPG data segments for each subject was 290, and the 18 time-domain and frequency-domain PPG features were computed for each segment (note that we computed the average value of each time-domain feature for each segment). The GSR data for each acupoint were sampled for five seconds. For each acupoint, only the median of the sampled GSR data was recorded as the feature point. The computed PPG and GSR features were used as the input for the neural network. A fully connected neural network (FCN) was implemented with Keras, where the input layers had 42 neurons. In other words, each subject provided 290 samples. Each sample contained 42 features (18 of them were extracted PPG features, and the other 24 were from the GSR data). Since for each subject we only measured GSR data once for each acupoint, the GSR features were the same in all 290 samples for the same subject. We employed a five-fold cross-validation to select the training data. In other words, in each experiment, data from 64 subjects were used for the training, and data from the other 16 subjects were used for testing. In the testing group, eight subjects had a “wiry pulse,” and the other eight subjects did not. The age range for the subjects who did not have a wiry pulse ranged from 15 to 96 (mean ± SD: 55.06 ± 19.2) years including 22 males and 18 females (BMI: 23.29 ± 2.42). The subject group that did not have a wiry pulse ranged in age from 13 to 85 (mean ± SD: 59.52 ± 15.28), including 16 males and 24 females (BMI: 23.87 ± 4.3).

To determine the level of importance of different features, we also adjusted the input layer of the neuron network for different sets of features, as shown in Table 2, where the GSR or PPG features were used alone. The prediction accuracy was generally less than 80%. However, when we combined both features together, the accuracy increased to 91%, which shows the benefit of using data fusion for TCM pulse type identification.

In addition, we evaluated the importance of specific features through random decision forests [24]. As shown in Table 3, the top three features with the highest weight in terms of classifying the wiry pulse were feature 2 and feature 3 in the PPG time-domain and the amplitude of the second harmonic in the PPG frequency-domain data. Specifically, subjects who had a wiry pulse tended to have a larger angle for the systolic peak, and their diastolic peaks were usually closer to the systolic peak over time. In addition, their second harmonics, corresponding to the kidney meridian, were often weaker as compared to the control group (i.e., the group that did not have a wiry pulse).

Finally, to further validate our results, we recruited another 10 subjects with a wiry pulse. Again, the determination of the pulse type was based on the joint agreement of three experienced TCM doctors. We use the previously trained model (based on data from the original 80 subjects) to classify these subjects. An accuracy of 90% was obtained in this experiment (i.e., the pulse type of one of these 10 subjects was not correctly predicted).

In our review of previous studies on pulse type prediction, most of them validated their results based on a small number of subjects. Some of the prior work also employed artificial neural networks (ANNs) for predicting pulse type. For example, Wang et al. [25] implemented a simple three-layer ANN to identify the wiry pulse and the slippery pulse. They reported an 87% predictive accuracy. Xu et al. [26] utilized seventeen features from the time-domain of pulse waves to train a three-layer ANN for predicting eight pulse conditions (not specified in their paper) and obtained 86% accuracy. As compared to these existing works, our study was based on a much larger and more diverse subject pool and thus had higher predictive accuracy.

## 4. Conclusions and Suggestions for Future Work

For several thousands of years, the pulse diagnosis has been one of the important diagnostic methods in traditional Chinese medicine (TCM). While a significant amount of effort has been put into the development of pulse diagnostic instruments, they are generally single-probe sensors that can only obtain limited information about pulse characteristics. In this work, we propose a low-cost smartphone-based system to infer the TCM pulse type based on the concept of sensor fusion. Specifically, by combining PPG and GSR data collected from a smartphone and running the prediction on top of a neural network, we were able to detect the wiry pulse, a commonly seen TCM pulse type, with a high degree of accuracy.

As a feasibility study, our current results are quite limited since they are only based on a small set of people with one specific TCM pulse type. Originally, we tried to formulate the problem as a multi-class classification problem. However, given that we did not have sufficient data for the “slippery pulse,” the results were poor. Therefore, we grouped the subjects with a “normal pulse” and “slippery pulse” together into a “non-wiry pulse” class, and only performed a binary classification in this study. However, we believe that our results can be generalized to infer other TCM pulse types, and we are currently working toward this goal by recruiting more subjects with different TCM pulse types (such as the slippery pulse, which is commonly seen in pregnant women). In addition, establishing an agreement of a TCM pulse type among different TCM practitioners is challenging. In this work, we evaluated our prediction results based on the opinion of a small panel of three TCM doctors. A quantifiable traditional Chinese medicine (TCM) pulse diagnostic model and its validation is another related topic we aim to investigate in the future. Finally, in this work, we computed the PPG and GSR data features, which were then used as inputs for a simple three-layer ANN to predict the wiry pulse. Recently, various deep neural network structures, such as RNN [27,28] and CNN [29], have been proposed for signal processing [30]. However, these deep architecture models generally suffer from over-fitting problems when the training data are small [31]. Due to the limited size of our dataset, we did not adopt these deep neural network models in this study. Nevertheless, we plan to investigate their applications to TCM pulse type prediction once we collect more data in the future.

## Figures and Tables

**Table 1:** PPG and GSR features used to estimate the traditional Chinese medicine (TCM) pulse type.

Time-Domain Features of PPG Data (As Shown in Figure 5)		
1	Pulse amplitude difference between pulse wave systolic peak (PWSP) and pulse wave diastolic peak (PWDP)	
2	Amplitude of pulse wave diastolic peak (PWDP)	
3	Angle of pulse wave systolic peak (PWSP)	
4	Pulse wave amplitude (PWA)	
5	Duration of systolic phase	
6	Duration of diastolic phase	
7	Pulse propagation time (PPT) between pulse wave systolic peak (PWSP) and pulse wave diastolic peak (PWDP)	
8	Inter-beat interval (IBI)	
Frequency domain features of PPG data		
the nth harmonic of base frequency (e.g., if the heartrate is 96 bpm, then C1 corresponds to 1.6 Hz, C2 corresponds to 3.2 Hz, …., etc.)	Corresponding TCM Meridian	
C1	Liver Meridian	
C2	Kidney Meridian	
C3	Spleen Meridian	
C4	Lung Meridian	
C5	Stomach Meridian	
C6	Gallbladder Meridian	
C7	Bladder Meridian	
C8	Large Intestine Meridian	
C9	San-yin-jiao Meridian	
C10	Small Intestine Meridian	
Skin impedance of acupoints from 12 meridians (including both left and right hands)		
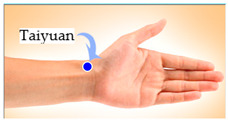 Taiyuan (LU9)	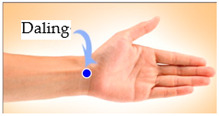 Daling (PC7)	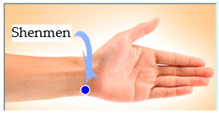 Shenmen (HT7)
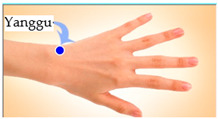 Yanggu (SI5)	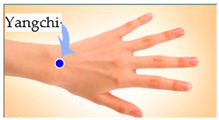 Yangchi (TE4)	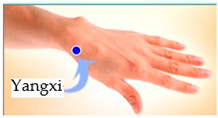 Yangxi (LI5)
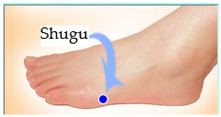 Shugu (BL65)	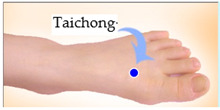 Taichong (LV3)	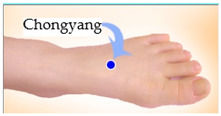 Chongyang (ST42)
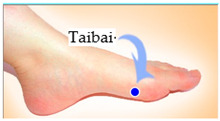 Taibai (SP3)	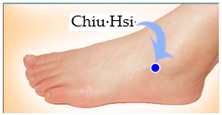 Chiu Hsi (GB40)	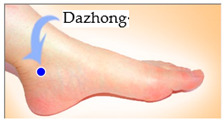 Dazhong (KD4)

**Table 2:** Comparison of feature accuracy using five-fold cross-validation.

Feature Used	Accuracy
Only 24 GSR features	62.5%
18 PPG (time-/frequency-domain) features	80%
8 PPG time-domain features	65%
10 PPG frequency-domain features	62.5%
8 PPG time-domain and 24 GSR features	72.5%
10 PPG frequency-domain and 24 GSR features	75%
18 PPG (time-/frequency-domain) features and 24 GSR features	91%

**Table 3:** Order of importance of features.

Feature	Weight	Wiry Pulse Group (mean ± std)	Non-Wiry Pulse Group (mean ± std)	*p* Value
PPG (2)	0.24636	2.92 ± 0.0303	1.534 ± 0.0838	<0.001 **
PPG (3)	0.225339	155.0205 ± 20.5951	47.8 ± 13.2765	<0.001 **
PPG-C2	0.214329	0.0046 ± 0.0049	0.0179 ± 0.0091	<0.001 **

** *p* < 0.001.

## Data Availability

The original data used to support the findings of this study are available from the first author upon request.
